# Supraglottic mucous membrane plasmacytosis: a case report and literature review

**DOI:** 10.1097/MS9.0000000000002766

**Published:** 2025-01-09

**Authors:** Sarah L. Gillanders, Thomas J. Crotty, James P. O’Neill

**Affiliations:** a Department of Otolaryngology-Head and Neck Surgery, Beaumont Hospital, Dublin, Ireland; b Royal College of Surgeons Ireland, Dublin, Ireland

**Keywords:** case report, head and neck, laryngology, larynx, mucus membrane plasmacytosis

## Abstract

**Introduction:**

Mucous membrane plasmacytosis is a rare, inflammatory disorder characterized by polyclonal plasma cells infiltrating the mucosal tissue of the oral cavity, oropharynx, laryngopharynx, or genitalia.

**Case presentation:**

We present a case of supraglottic plasmacytosis which presented a management challenge due to a complicated past medical history preventing oral steroid administration.

**Discussion:**

A literature review is conducted to determine alternate treatment options in for this challenging case and others. A discussion of diagnosis, treatment and aftercare follows the case report and literature review.

**Conclusion:**

This case report illustrates the rare condition of laryngeal mucous membrane plasmacytosis. Application of steroids to the specific site of pathology appears to be a promising management course.

## Introduction

Mucous membrane plasmacytosis (MMP) is a rare, inflammatory disorder characterized by polyclonal plasma cells infiltrating the mucosal tissue of the oral cavity, oropharynx, laryngopharynx, or genitalia^[1]^. Only a small number of cases have been reported in the literature and isolated supraglottic laryngeal involvement is exceedingly rare^[2]^. However, given the severity of symptoms and risk of airway compromise timely diagnosis and treatment is imperative. Multiple treatment options have been trialed, however, no high quality evidence exists^[3]^ and experts lack consensus^[4]^. We present a case of MMP of the supraglottic larynx which presented a management challenge due to a complicated past medical history. This case has been reported in in alignment with Surgical Case Report (SCARE 2023)^[5]^ guidelines.

## Case report

**Figure 1.**
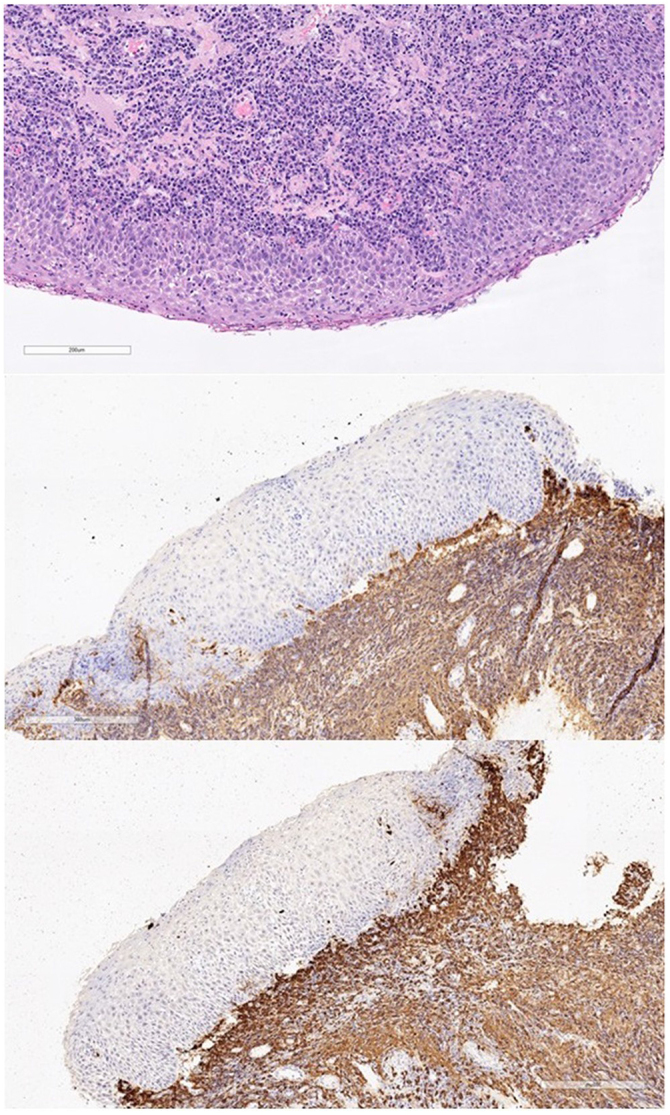
Arytenoid tissue infiltrated by abundant plasma cells, kappa and lambda staining.

**Figure 2.**
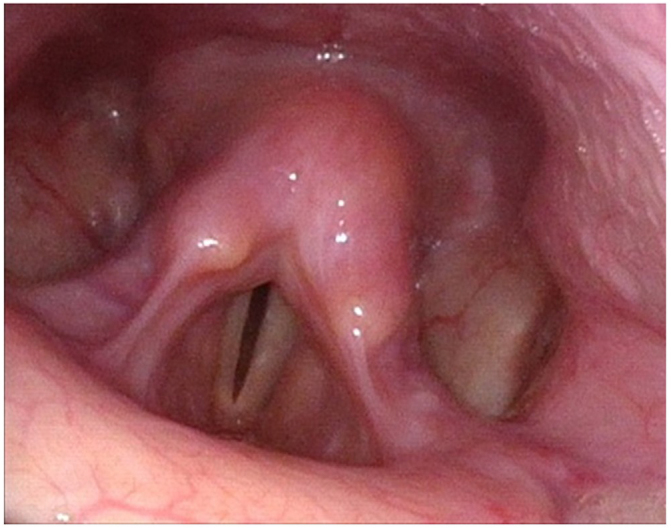
Post treatment flexible nasendoscopy clinical image.

A 62-year old women was referred to the outpatient otolaryngology clinic with an incidental finding of supraglottic swelling during an OGD. At time of review, she complained of globus pharyngeus, dysphagia to solid foods and fluctuating hoarseness. Notably, her past medical history was significant for pyloric stenosis secondary to peptic ulcer disease which required regular endoscopic dilatation and surveillance with tissue sampling. She had no other past medical or surgical issues, and no history of tobacco or alcohol use. Clinical examination with flexible nasendoscopy revealed diffuse edema and cobblestone mucosal thickening of the supraglottic larynx. The underlying vocal cords were mobile. The rest of her head and neck examination was normal. These findings prompted urgent laryngoscopy under general anaesthetic with tissue sampling. The patient underwent an uncomplicated procedure with multiple biopsies of the supraglottis and no airway issues post-operatively. The biopsies revealed an inflammatory process with polyclonal plasma cell infiltration without evidence of plasmacytoma or dysplasia. This was confirmed with both kappa and lambda staining (Fig. 1). Following multidisciplinary team (MDT) discussion, a diagnosis of MMP involving the supraglottic larynx was confirmed. In consultation with her primary gastroenterology team, oral steroids were contraindicated due to her underlying peptic ulcer disease. As a result, we opted to perform direct laryngoscopy and administer intralesional triamcinolone (40 mg/mL a total of 1 mL over 3 sites). The procedure was uncomplicated. She was discharged post-operatively with a prescription for budesonide (0.5 mg/mL) nebulizers twice daily. Her 2-week post-operative review revealed a major improvement in symptoms and decrease in laryngeal edema on flexible nasendoscopy. Following a literature review of aerodigestive tract MMP, a suggestion was made to review the histopathology from previous gastric and esophageal biopsies to out rule a missed diagnosis of gastric MMP. Following MDT discussion the diagnosis of two separate pathologies were confirmed. Six months following her operation clinical review revealed a normal appearing larynx on flexible nasendoscopy (Fig. 2) and the patient was asymptomatic. At the time of her last review, 1 year post treatment the findings are unchanged. We plan to continue with 6 monthly follow-up or as required should symptoms arise.

## Methods of literature review

Using the RCSI library resources, we searched the following electronic databases to identify reports of relevant papers: MEDLINE PubMed, The Cochrane Central Register of Controlled trials (CENTRAL), Ovid MEDLINE, EMBASE, and grey literature. Key search terms of mucus membrane plasmacytosis, plasma cell mucositis and idiopathic plasmacytosis were used. Initially we included any study that reported mucosal membrane plasmacytomas of the aerodigestive tract in adults. Studies were limited to those published in the English language or those with a full English translation available. Studies involving areas other than the aerodigestive tract or were histology was not confirmed were excluded. Following a review of included bibliography additional papers were included. Finally papers that did not include laryngeal involvement were excluded. Table 1 represents a summary of the laryngeal cases identified and treatment options reported.

**Table 1:** Summary of reported cases of laryngeal mucous membrane plasmacytosis.

Author	No.	Site	Treatment	Response	Follow-up years
Bharti, 2003	1	Larynx + oral (buccal)	Topical and systemic steroids, antifungal	Improvement	1
Coppola, 2022	3	Larynx + oral/oropharyngeal	Oral and topical steroids, immunosuppressive agents, debulking procedures	Improvement	N/A
El Naderi, 2013	1	Larynx	Interoperative steroid injection	Improvement	4
Ferreiro, 1994	9	Larynx + oral (buccal + lip)	Oral steroid, antibiotics and surgical resection, tracheostomy	Progressive	11
Fogarty, 2001	1	Larynx + oral (tongue)	Topical and oral steroids, Radiotherapy	Improvement	21
Khan, 1997	1	Larynx	Topical steroids (beclomethasone oral spray, Corsodyl mouthwashes)	Improvement	1.75
Makarenko, 2020	1	Larynx	Oral steroids	Improvement	15
McLennan, 2023	1	Larynx	Topical steroids (inhaled budesonide)	Remission	2
Mistry, 2012	1	Larynx	Surgical—tracheostomy, balloon dilatation	Improvement	0.75
Nakamura, 2022	1	Larynx + oral (buccal) + nose	Oral steroids	Improvement	0.25
Smith, 2018	1	Larynx	Oral and topical steroids	Improvement	0.5
Timms, 1991	3	Larynx + oral (buccal + lip)	Oral and topical steroids	Remission	7
Triplett, 2018	1	Larynx	Oral, mycophenolate mofetil, surgery	Improvement	2
White, 1986	1	Larynx + oral (buccal + lip)	Conservative—exposure to dentures removed	Improvement	9

## Discussion

### Summary of results

A total of 37 studies were identified that met criteria for inclusion affecting the aerodigestive tract. Of these studies, 14 studies^[1-4,6-15]^ reported laryngeal involvement from 26 patients, with only seven of these cases affecting the larynx in isolation.

### Diagnosis

Clinical presentation of mucous membrane plasmacytosis is dependent on the location of the aerodigestive tract which it affects. MMP of the oral cavity presents as painful, erythematous and swollen lips and gums rarely associated with ulceration^[6]^. Nasal MMP presents as a painful mass in the nasal cavity and may cause obstruction and nasal discharge^[16]^. Pharyngo-laryngeal MMP causes symptoms of pain, globus, dysphonia and dysphagia. More advanced cases can cause difficulty breathing and stridor. On examination the mucosa is often described as a diffuse cobblestone appearance with significant oedema^[8]^. MMP is rare, while it should be included in differential diagnosis of these findings infectious and malignant pathologies are more likely and must be excluded. As a result tissue biopsy is essential for formal diagnosis. Characteristically, histopathological analysis demonstrates a diffuse and expansive subepithelial polyclonal mature plasma cell infiltrate with no anaplasia or prominent nucleoli^[8]^. A variety of epithelial appearances have been described. The most commonly reported description is hyperplastic, acanthotic epidermis with narrow and elongated rete ridges. However, an atrophic, eroded and ulcerative epidermis have been described^[6]^.

### Treatment

Many treatment options have been described in the literature with varying degrees of success. Simple conservative methods of withdrawal of irritant substances with clinical surveillance have been successfully reported by several authors^[15,17-20]^. Medical treatment most widely reported involved some form of corticosteroid administered either topically^[4,7,10,16,18,21,22]^ or orally^[1,2,6,8,9,13,23,24]^. However, results have been inconsistent. Only one author reported intralesional steroid injection^[3]^ similarly, to our case this method resulted in rapid improvement and remission during the follow up period reported. Other medical treatments offered include antifungal^[6,25]^, antibiotic^[8,24]^, and immunosuppressive agents^[7,18,21,24]^. One author reported successful treatment with the biologic agent adalimumab^[24]^ after failure of other medical treatments. Surgical resection and tracheostomy has also been required for management in some progressive cases^[7,8,11,21]^. Debulking procedures have shown improvement in symptoms initially but in all cases have not stopped disease progression or induced remission. Additional methods reported include C02 laser resurfacing^[21]^, balloon dilatation^[11]^, cryotherapy^[26]^ and low dose radiotherapy^[9,21]^. Consensus on optimal treatment remains elusive.

### Follow-up

In most cases follow up was limited, however several papers reported a ten to twenty year follow up period^[1,8,9,21,25]^. The clinical course of MMP is described as waxing and waning and associated with long periods of remission. However, Makarenko et al. also warn of the possibility of disease progression to loss of organ functions in treatment-resistant cases. In one case reported by Pepper et al oral plasmacytosis was subsequently diagnosed with squamous cell carcinoma of the lip^[21]^. This was twenty years post plasmacytosis diagnosis following numerous medical treatments such as tacrolimus, methotrexate, CO_2_ laser and on a background of a significant smoking history. As such Pepper et al cautioned the use of immunosuppressive agents for treatment of plasmacytosis in patients with other risk factors for malignancy^[21]^.r

### Strengths and weaknesses

Case reports inherently are associated with additional bias not linked to other research associated more rigorous scientific methods. Although there are the obvious limitations with this case report it is worthwhile in adding to the lacking body of evidence for this rare condition, as well as providing awareness for similar cases and highlights an area of research development for future treatment options.

## Conclusion

In conclusion, this case report illustrates the rare condition of laryngeal mucous membrane plasmacytosis. Timely diagnosis with tissue sampling is crucial to out rule a more sinister pathology. Despite the lack of evidence or consensus on treatment steroids are the most commonly utilized. Our patient demonstrated a major improvement following inhalation and intralesional steroid injection. Administration of steroids to the specific site of pathology appears to be a promising management course.

## Data Availability

Not applicable.
